# Evaluation of statistical methods applied in theses and dissertations in an Open, Distance and e-Learning University

**DOI:** 10.1371/journal.pone.0319654

**Published:** 2025-03-31

**Authors:** Legesse Kassa Debusho, Mahlageng Retang Mashabela, Phuti Naphtaly Sebatjane, Sthembile Sithole, Busisiwe Tabo, Eeva-Maria Rapoo

**Affiliations:** Department of Statistics, College of Science, Engineering and Technology, University of South Africa, Johannesburg, Republic of South Africa; Instituto Tecnologico Autonomo de Mexico, Mexico City, MEXICO

## Abstract

The appropriate application of research methods and statistical analyses used in the studies directly affects the quality of scientific studies. Due to the possibility of employing an incorrect statistical technique, it is crucial to choose a statistical method based on the study’s data and research objectives. This study aimed to evaluate whether statistical techniques applied in the theses and dissertations were appropriate for planning surveys or experiments and analyzing data, and to identify common mistakes that master’s and doctoral students made when using statistical techniques for the intended goals. The study reviewed 139 master’s theses and doctoral dissertations submitted to seven agricultural and environmental sciences disciplines at a leading Open, Distance and e-learning university in Africa between 2015 and 2020. These dissertations and theses used mixed and quantitative research methods. The analysis of variance test was the most often used statistical test, according to the results, followed by the student *t*-test and the Chi-square test. At least one blatant methodological error was found in 41.0% of theses and dissertations, either in the data collection process or in the data analysis. Examples of these errors include the use of a simple random sampling technique despite the heterogeneous population units, the conversion of count responses to binary responses and percentages for fitting logistic and general binomial regression models, and the incorrect modeling of correlated data using generalized linear models. The results of this study will create greater awareness of the common errors that postgraduate students make when using statistical methods to design experiments or sample surveys and analyse data. In addition, the findings inform the university management to plan for specific training in statistical methods appropriate for a range of academic fields.

## Introduction

Statistics is an integral part of scientific research, from the design of an experiment or survey to the presentation of results, and affects all aspects of the research process, from data collection and management to analysis and interpretation. The quality of scientific studies is directly related to the proper application of research methods and statistical analyses employed in the studies [1]. This is why it is important to select a statistical method based on the research objectives and study data, as there is a risk of using an inappropriate statistical approach that may lead to conclusions that are considered questionable by the academic’s community [2,3]. However, there is substantial evidence in the literature [4–6] of poor reporting and use of statistical methods in master’s theses and doctoral dissertations. Govil, Qasem, and Gupta [7] evaluated PhD theses in the social sciences disciplines for the suitability of statistical methods used in the data analysis. They found that, out of the theses that were evaluated, 87% had improper sample selection, 53% did not meet the assumptions of the statistical methods used for analyses, 24% did not use the proper tool according to the variable requirements, and 21% had errors, such as using the incorrect method concerning sample size and number of variables. They also reported that while most researchers discussed random sampling, few carried it out.

Furthermore, there were similar problems observed in published articles [3,8–10]. For example, in a study using 292 plant science articles published between 2000 and 2006 in the Brazilian quality journal Qualis A, Bertoldo et al. [11] noted that the authors had diﬃculty selecting appropriate statistical methods with the chosen experimental design. Among the papers that examined more than one factor, the mean comparison methods used in three-quarters of them were considered inadequate due to the misuse of mean comparison tests. In contrast, 22% and 3% were classified as appropriate and partially appropriate, respectively. Lee [12] reported that over half of the articles published in biomedical journals wrongly applied statistical methods. In a census of 307 articles published in the Archives of Veterinary Science between 2000 and 2010, Montanhini Neto and Ostrensky [2] assessed the use of statistical methods, and they found that in over a third of the articles, the methods used in the analyses were inadequate. Similar results were also reported by Junior et al. [3]. In line with Glickman et al. [13], inadvertent mistakes may also stem from a lack of planning, particularly when researchers consider statistical analysis only after obtaining experimental data.

However, research that assesses the application of statistical methods in master’s theses and doctoral dissertations using data from open, distance e-learning institutions remains quite limited. The authors of this study have been observing various problems in statistical methodologies in data collection or conducting sample surveys, in designing experiments and in methods for data analysis in the research proposals of master’s and doctoral students reviewed recently during ethical review committee meetings in the disciplines of Agricultural and Environmental Sciences in the leading Open, Distance and e-Learning University in Africa. These include, for example, planning to collect data without any consideration of the statistical validity of the experimental or survey design; proposing a simple random sampling technique for a heterogeneous population; suggesting inappropriate statistical methodologies for data analyses (e.g., proposing linear models, either ordinary analysis of variance or linear regression models) to analyse data that will be collected from longitudinal studies as though observations are independent. Such errors could produce erroneous results or superficial interpretations, which would lead to incorrect inferences being made from the data. In addition, misuse of statistical techniques in scientific investigations violates ethical standards and potentially deceives aspiring researchers [14,15].

While the authors’ of this paper experiences with statistical support have been more widespread at the proposal stages starting in 2019, their observation makes them believe that similar issues may have arisen in the past regarding the quality of both design and statistical analysis applied in postgraduate students’ theses and dissertations. Postgraduate students must be knowledgeable about research and statistical techniques to conduct high-caliber research, comprehend research publications well, or do both. Data regarding the application of statistical methods in the research practices of past graduates in various disciplines may offer some relevant recommendations for potential interventions, should they be needed. This study’s goal was to assess the statistical techniques used in master’s and doctoral theses and dissertations in fields related to agricultural and environmental sciences that were submitted between 2015 and 2020. In particular, the study evaluated whether statistical techniques applied in the theses and dissertations were appropriate for planning surveys or experiments and for analyzing data. It also identified common mistakes that graduates made when applying statistical techniques for the intended goals. The focus was on the statistical methods without considering the subject matters investigated in a thesis or dissertation.

## Materials and methods

### Data

The study sample consisted of 139 master’s theses (62%) and doctoral dissertations (38%) from five disciplines of agricultural and environmental sciences of a leading Open, Distance and e-Learning (ODeL) university in Africa (see Table 1). These theses and dissertations have been submitted between 2015 and 2020.

**Table 1:** Distribution of theses and dissertations by degree, disciplines and year of graduation.

Degree	Discipline	Year						
		2015	2016	2017	2018	2019	2020	Total
	Agriculture	1	1	0	0	0	0	2
	Consumer sciences	0	1	1	4	4	5	15
	Environmental management	12	0	3	2	4	3	24
MSc	Environmental sciences	2	0	3	6	2	5	18
	Geography	4	0	1	1	1	2	9
	Life science	5	2	1	2	2	3	15
	Nature conservation	0	0	0	0	3	0	3
	Total	24	4	9	15	16	18	86
	Agriculture	0	0	2	3	2	1	8
	Consumer sciences	0	2	2	0	0	1	5
	Environmental management	1	0	0	5	2	6	14
PhD	Environmental sciences	0	0	0	3	3	6	12
	Geography	3	2	0	1	3	2	11
	Life science	0	0	1	0	1	1	3
	Nature conservation	0	0	0	0	0	0	0
	Total	4	4	5	12	11	17	53

A comprehensive search was done on the institutional repository to identify relevant theses and dissertations for this study. The theses and the dissertations assessed in this study were accessed from the institutional repository between 3 January 2022 and 16 March 2022. Doctoral students were involved in downloading each discipline’s theses and dissertations submitted between 2015 and 2020 from the repository. All authors participated in reviewing the abstract and methodology section or chapter of every thesis and dissertation to select or exclude it based on the inclusion/exclusion criteria (presented in the next section) to compile the final sample. Since there was no primary hypothesis that the research team wanted to test in this study, there was no need to formally power the study or to state expected differences between parameter differences (or margin of error) to determine the sample size using formal statistical considerations [16].

**Fig 1:**
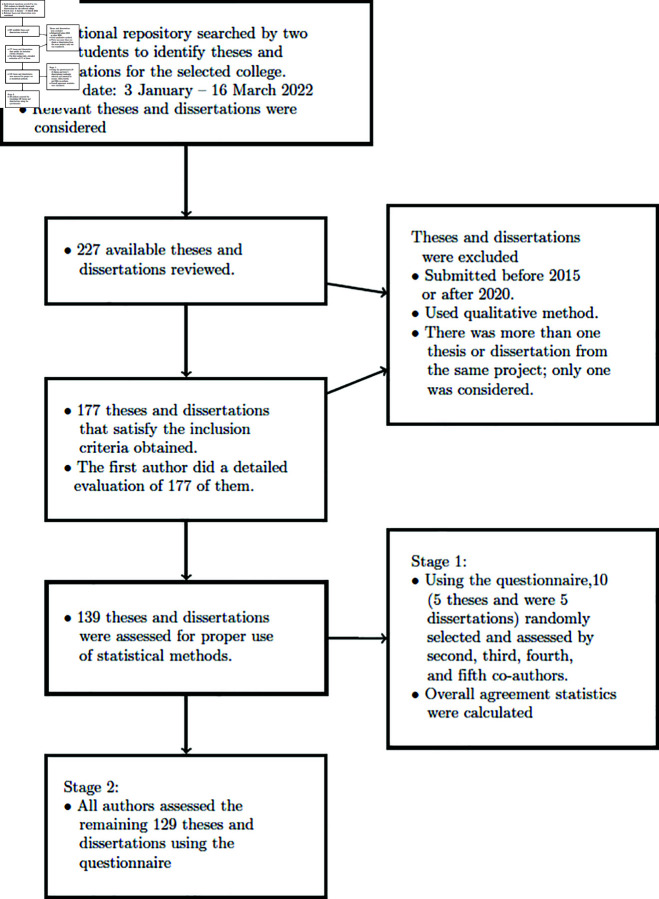
Flow diagram summarising the stages for assessment.

### Inclusion and exclusion criteria

All relevant theses and dissertations submitted between 2015 and 2020 that applied at least one statistical method either to design an experiment or sample survey or to analyse the research data were included in the study (Fig 1). Since the study has considered only theses and dissertations that applied either quantitative methods or mixed methods in their research, those that used only qualitative methods were excluded from the study. The focus of this study was on the statistical methods without considering the subject matters investigated in a thesis or dissertation. The selection of a thesis or dissertation from any single research project, where there was more than one student involved in a particular study or dataset, was limited to one student. This restriction was introduced to ensure that the survey results were not disproportionately influenced by the bad or good practices of one.

### Assessment of theses and dissertations

The 139 theses and dissertations were assessed for proper use of statistical methods using the questionnaire developed by the research team. The questionnaire asks whether a thesis or dissertation has research objective(s) and hypothesis(es) or not, about the type of experimental design or sample survey and if they were correctly or appropriately applied, about the number of replications/sample size, data completeness, statistical methods and assumptions, diagnostics to assess the assumptions, and if the assumptions were not met whether remedial actions were taken or not.

Every thesis or dissertation was read by all the authors, the assessment undertaken independently and then a questionnaire filled out. There were two phases to this assessment. In stage 1, a random sample of ten theses and dissertations (five from each category) was evaluated independently by the second, third, fourth, and fifth co-investigators. The data generated were captured in a table format to assess the reliability of the co-investigator assessments. The agreement among the co-investigators assessments was examined by applying the mean Kappa statistic and reliable coeﬃcient. The two statistics were calculated using relevant functions from the psych [17] R package. In stage 2, all investigators assessed the remaining theses and dissertations. Those disagreements or differences in interpretation of the checklists or questions were resolved by discussion with the first and sixth authors. Where necessary, the relevant theses or dissertations were reanalysed by the research team.

The correctness, robustness, eﬃciency, and relevance of the statistical methods applied in selected theses or dissertations were assessed using a yes or no assignment for each question based on the statistical concepts presented in the literature [3,8,10,17–20]. In the current study, correctness refers to whether the statistical method applied in a thesis or a dissertation was appropriate. For instance, it is wrong to use a *t*-test to make statistical inference on a continuous variable that has a skewed distribution and the sample size is small. Many statistical methods rely on several assumptions, e.g., normality, independence etc. If those assumptions are incorrect, the selected method can produce misleading results. In this context, we described the chosen method as lacking robustness. A statistical method rated is ineﬃcient if, for example, a nonparametric rather than a parametric method is used for a statistical inference where a continuous response variable has a symmetric distribution. Finally, an analysis was regarded as relevant if it answered the question posed in the study. For instance, multivariate technique such as a principal components analysis might be correct and eﬃcient for summarising a multivariate dataset but might have had no bearing on the stated objective of a thesis or dissertation.

### Ethical consideration

Permission for the study was obtained from the School of Science Ethics Committee (ERC Reference Number: 2021/CSET/SOS/060). In addition, permission to access completed theses and dissertations (Reference Number: 2021_RPSC_040) in the public domain in electronic format via the Institutional Repository was obtained from the Research Permission Subcommittee (RPSC) of the University Senate, Research, Innovation, Postgraduate Degrees, and Commercialisation Committee (SRIPCC), therefore informed consent was not obtained from the study participants.

## Results and discussion

The findings obtained from the questionnaire-based assessment of theses and dissertations, and discussion are presented below.

### Reliability of the questionnaire

For the random sample of ten theses and dissertations assessed at stage 1, the mean Kappa statistic and reliable coeﬃcient were 0.88 and 0.83, respectively, indicating good agreement among the co-investigators and the reliability of the questionnaire.

### Academic discipline

**Fig 2:**
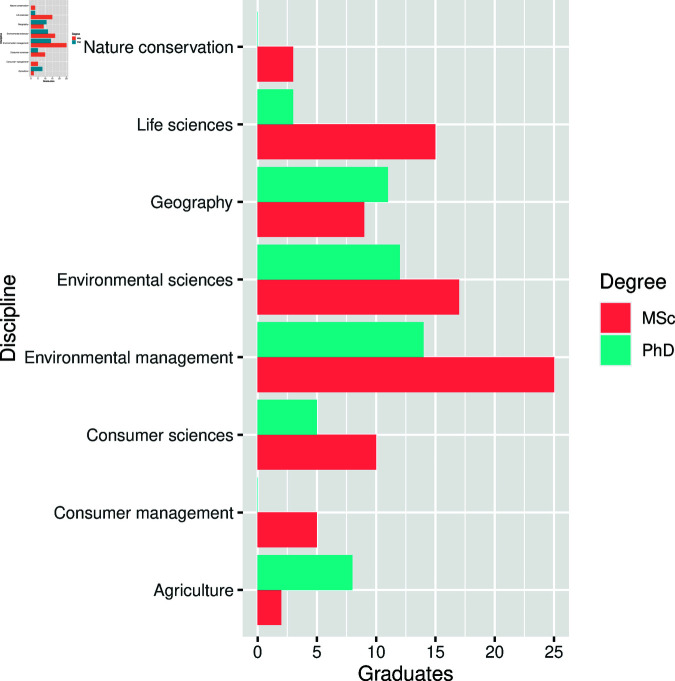
Distribution of type of degrees by academic disciplines.

As displayed in Table 1, in total 139 theses and dissertations were considered for assessment, of these 86 (62%) were master’s theses. The Department of Environmental Sciences had the highest percentage of master’s theses (42, 48.8%), with 25 coming from the field of Environmental management and 17 from Environmental sciences (see Fig 1a), which was followed by the field of Consumer sciences (15, 17.4%; where 10 of them from the field of Consumer sciences and 5 from Consumer management), and Life sciences. The lowest numbers of master’s theses belonged to the field of Agriculture and Animal Health (2, 2.3%). Similarly, the highest number of doctoral dissertations were from the discipline of Environmental Sciences (26, 49.1%, where 14 of them from Environmental management and 12 were from the field of Environmental sciences, see Fig 2), which was followed by the Department of Geography (11, 20.8%) and there was no doctoral dissertation submitted in Nature Conservation between 2015 and 2020.

**Fig 3:**
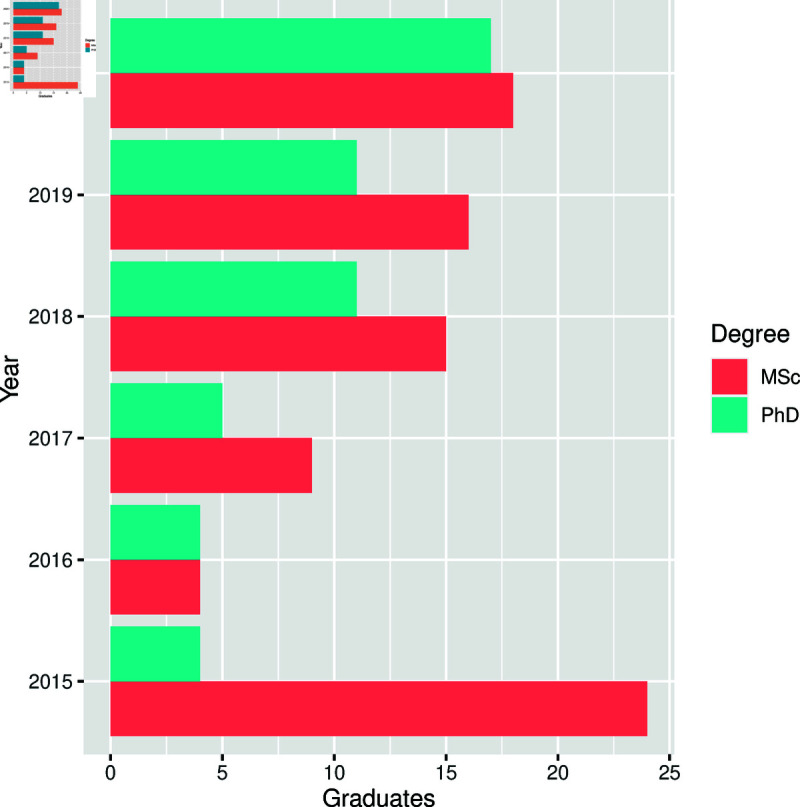
Distribution of type of degrees by year.

The distribution of graduates by year is shown in Fig 3. The findings indicate that between 2016 and 2020, there was an increase in the number of MSc and PhD graduates. Over time, there has consistently been more MSc graduates than PhD graduates.

### Research methods, experimental or survey design

All the theses and dissertations had well-structured research objectives and hypotheses. The research methods used in the theses and dissertations considered in the current study were grouped into two categories, namely quantitative and mixed methods, based on the inclusion criteria. The distribution of these categories by degree type is shown in Table 2.

The results in Table 2 show that among 139 theses and dissertations assessed in the disciplines of Agriculture and environmental sciences between 2015 and 2020, 120 (86.3%) of them specified the type of research methods applied in their studies. Of the 120, 61 (50.8%) are used the quantitative method.

**Fig 4:**
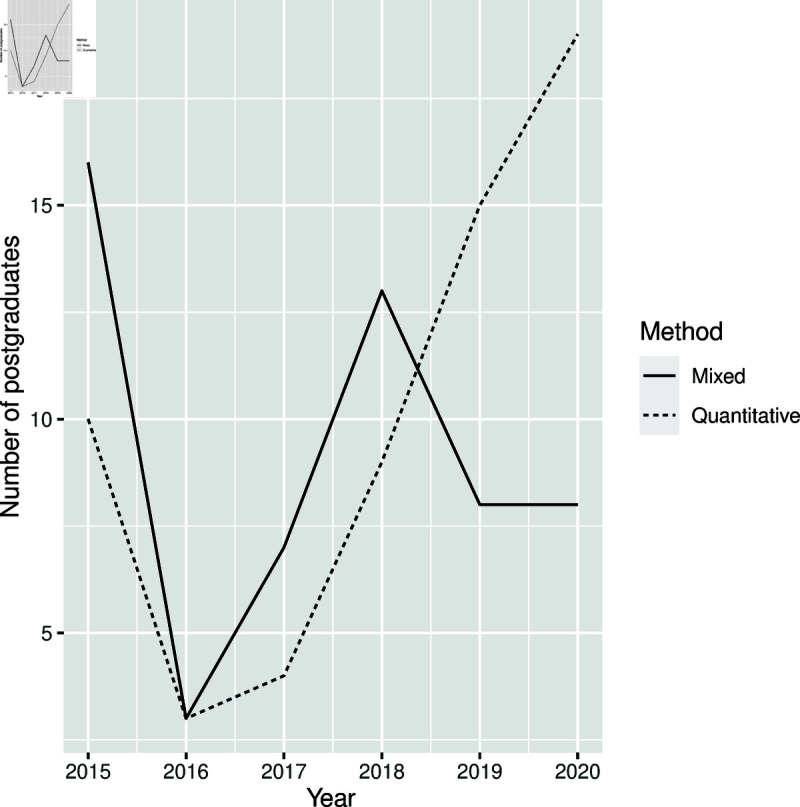
Distribution of research methods by years.

Fig 4 demonstrates that in 2015, more theses and dissertations applied the mixed method than the quantitative method, but the numbers dropped for both methods in 2016 and increased in 2017 and 2018. In 2019 and 2020, the number of theses and dissertations which applied the quantitative method increased compared to the number of theses and dissertations which applied the mixed method. Ninteen of them assessed in this study were not included in Fig 4 because the type of research methods used were not stated.

Out of the ten theses and dissertations from the fields of agriculture and animal health that were included in the study, two MSc theses and three PhD dissertations used a sample survey approach, while two PhD dissertations from the field of agriculture and one MSc thesis from the field of consumer sciences used a complete random design (CRD). A response surface design was used in two MSc theses from consumer sciences, an augmented design was used in one MSc thesis from life sciences, a diallel design was used in a PhD dissertation from agriculture, a randomized complete block design (RCBD) was applied in a PhD dissertation from agriculture, and in two MSc theses from environmental sciences. The findings indicate that only 40.4% of all theses and dissertations correctly used sampling techniques or experimental designs. Of all the theses and dissertations assessed, two of them had only one treatment group, and treatment group was not applicable in 58 of them. There were 41 of them with 2–4 treatment groups but only three of them considered a factorial treatment structure, only eight from 38 of them which had five or more treatment groups considered a factorial treatment structure and these eight have also considered interaction effect(s).

### Experimental or sampling unit and randomization procedure

**Fig 5:**
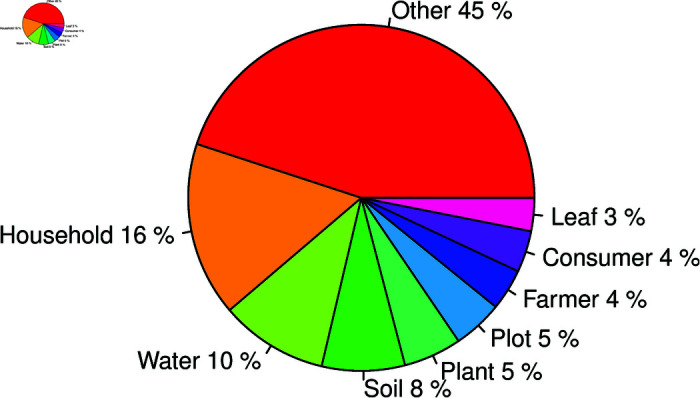
Distribution of experimental or sampling units in theses and dissertations.

A sampling unit is an object or animal/patient that can be selected (with known probability) from a population from which measurements were taken during the sampling process. It is the minimum unit of observation that possesses the properties being studied. Selection of a unit from a population as a sampling unit depends on who a target group is and what data are available about the population. The experimental unit is a physical object, e.g., plot of land, seed, household, animal etc, assigned to a treatment or intervention. It is the unit of statistical analysis, and before running a survey or an experiment, it needs to be properly identified. Failure to correctly identify it may lead to incorrect inferences from a study [21]. In 129 (92.8%) of the theses and dissertations, the experimental or sampling units were identified but in ten of them, the units were not clearly identified. According to Fig 5, the most frequently used experimental/sampling unit was household 21 (16%), followed by water 13 (10%) and soil 10 (8%). The category “other” contains over 30 different types of experimental/sampling units.

**Table 2:** Research methods by type of degrees.

Method	Degree		
	Master	PhD	Total (%)
Quantitative	46	15	61 (43.9)
Mixed	29	30	59 (42.4)
Not stated	11	8	19 (13.7)
Total (%)	86 (61.9)	53 (38.1)	139

**Fig 6:**
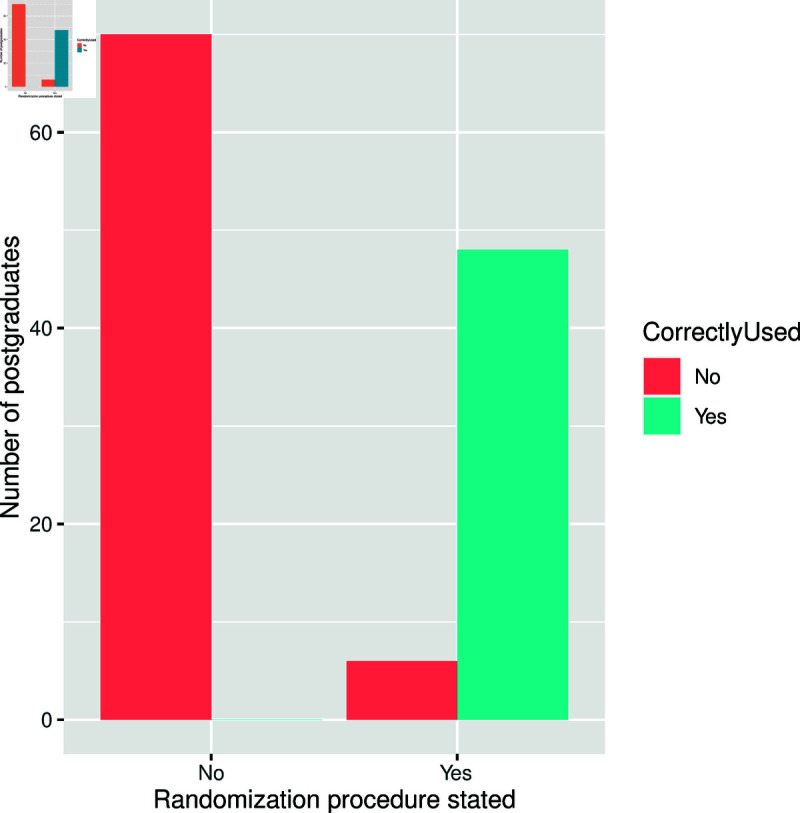
Distribution of theses and dissertations that correctly used the randomization procedure as stated.

A randomization procedure was stated only in 54 of the theses and dissertations, but it was applied as stated in the methodology chapter or section, i.e., correctly, in 48 (88.9%) of them, see Fig 6.

### Number of replications and sample size

Replication is one of the three principles of experimental design (where the other two are randomization and local control) and allows estimation of experimental errors. Consequently, the accuracy of an experiment and treatment estimates depends on the number of replications, where the accuracy improves with increasing replication counts. It is not feasible to increase the number of replications in experiments with high number of treatments, though, because the size of the experimental area, the biological material, and the financial resources are limiting factors to determine the number of replications [3]. There were 11 theses and dissertations which used the design of experiments. The number of replications was justified in the methodology section only in one MSc thesis from the Environmental sciences discipline. Except in one PhD dissertation from the Agricultural sciences discipline, in the other ten dissertations, there was no calculation or formulae given to justify the number of replications and used equal replications per treatment group. In the PhD dissertation, where there was an unequal number of replications per treatment group or level, there was no justification why unequal number of replications per treatment group was used.

The sample size was justified in the methodology section/chapter of 14 (10.9%) of 128 theses and dissertations which did not use an experimental design. In each of 11 of these 14, a specific calculation was given to justify the sample size. Although various methods are available depending on methods of sampling, parameters of interest in statistical inference, or type of response variables, in most of the theses and dissertations, the sample sizes were calculated either using tables available online or from literature or using a simple random sampling sample size formula for proportion without checking the homogeneity of a target population or type of response variable/parameters of interest in the statistical inference. In some cases, 10% of the population was taken as a sample without any justification. Therefore, the methods applied for sample size calculations in these theses and dissertations were incorrect or ineﬃcient. In 17 (13.3%) of 128, there were an equal number of sampling units either per stratum or group. Only in two PhD dissertations from environmental sciences and geography disciplines, that the unequal sample sizes per stratum or group was justified.

### Data completeness

In a study missing data occur due to various reasons, for example, missing data occur in a longitudinal study due to unexpected reasons such as an animal dying before completing the study, a plot of land may be damaged before harvesting, a household may change address etc. Generally, missingness in a study data should be documented and reported as to why the data was missed. The data may be missing completely at random (MCAR), missing at random (MAR), or missing not at random (MNAR). In MCAR, missing data are randomly distributed across a variable and unrelated to other variables in the study or unobserved variables; in MAR data are not randomly distributed but they are accounted for by other observed variables, whereas in MNAR, missing data systematically differ from the observed values and values are missing for reasons related to observed values. In MNAR, the collected sample data may not be representative of the study population. Missingness that are MCAR or MAR are ignorable because they do not differ from the observed values, however in MNAR since the missing data systematically differ from the observed values, they are non-ignorable. A multiple imputation is advised to handle missing data when, as a rule of thumb, the proportion of missing data is above 5% [22] or below 40% of the data; data are missing on both dependent and independent variables or independent variables [23]; and if the MCAR or MNAR assumption is not plausible [24].

Missingness of data was reported in 42 (30.2%) of theses and dissertations either for a complete sampling/experimental unit or a single observation of a variable within a sampling unit. In five of these, data were either intentionally omitted or not collected and in 17 of them the reasons for missing data and why data were not collected were not explained. Of those 42 reported missing data, 39 of them did their statistical analyses using complete cases, two of them imputed missing data, one applied the *k*-nearest imputation method and in the second one the author used statistical software to fill in the missing observations but did not specify the method that was applied, and in one of them there was no discussion how the missing observations were filled.

### Statistical methods

For the data analysis of this study, all the statistical methods in every thesis or dissertation were recorded. However, if a statistical method was used more than once in the same thesis or dissertation, it was counted only once. The statistical methods applied for data analysis in the theses and dissertations considered in this study are presented in Table 3. Results in the table show that descriptive statistics was applied in 112 (80.6%) of them. This was followed by ANOVA or MANOVA, correlation analysis, and regression analysis which were employed in 45 (32.4%), 43 (30.9%), and 34 (24.5%) of theses and dissertations, respectively.

**Table 3:** Statistical methods used in theses and dissertations.

Method	Number of theses & dissertations (%)
Descriptive statistics	112 (80.6)
ANOVA or MANOVA	45 (32.4)
Correlation analysis	43 (30.9)
Regression analyses	34 (24.5)
Chi-squared test	30 (21.6)
Multivariate methods	26 (18.7)
*t*-test	21 (15.1)
Spatial and spatiotemporal analysis	9 (6.5)
Model diagnostics	6 (4.3)
Non-parametric tests	3 (2.2)
Other	13 (9.4)

The regression analyses include general and generalized (binary/ordinal/multinomial logistic, Poisson regression models) linear models. The multivariate methods include principal component analysis (PCA), exploratory factor analysis, structural equation modelling, cluster analysis, discriminant analysis, canonical correspondence analysis, canonical redundancy analysis, ordination analysis, and orthogonal projection. The most commonly applied multivariate method in this study was PCA followed by factor analysis. The non-parametric tests include the sign, the Wilcoxon sign, and the Kruskal–Wallis tests. The category “Other” includes time series analysis, machine learning methods, generalized additive model, linear mixed model, two-stage least square regression model, and Kappa coeﬃcient.

To make correct statistical inferences, a researcher should select an appropriate statistical method for data collection and analysis. In this study, from 139 theses and dissertations 90 (64.7%) of them, either did not use the appropriate study design or did not eﬃciently use the data they have collected. In 57 (41.0%) of them, we have observed at least one clear methodological error either in the data collection procedure or in the data analysis. In 21 of these where a *t*-test was employed, only one of the theses assumed that the response variable was approximately normally distributed or used a robust approach, but in the other 20, either the authors neglected to address the normality assumption or the assumption was violated and no remedial action considered.

After identifying the response variables in appropriately conducted experiments, analysis of variance (ANOVA) is one of the most commonly used statistical methods for analyzing data from agricultural and biological research. The results of the ANOVA can be used to determine whether the effects of treatments and blocks on response are significantly different or not. However, it is necessary to check whether the data meets the assumptions of ANOVA, i.e., homogeneity of variances, independence and distributions of random errors [25]. Although reported results show that ANOVA is the most widely applied method in agricultural and animal sciences experiments [2,3], in the current study, ANOVA was used only in 32.4% of the theses and dissertation. None of the 45 theses and dissertations where ANOVA or MANOVA was used had any evidence of heterogeneity or homogeneity of variances and only in two of these data the response variable was assumed to be approximately normally distributed. Similar to the *t*-test, in the other 43, either the authors neglected to address the normality assumption or the assumption was violated, and the authors considered no remedial action; hence, the ANOVA or MANOVA applied in these theses and dissertations could lack robustness. Moreover, in six of them, parametric ANOVA was used for ordinal responses, hence they applied an ineﬃcient method. Similarly, in none of the 34 theses and dissertations where linear regression analyses were applied, there was no evidence of constant variance for the residuals and residuals were not assumed to be approximately normally distributed or assessed for normality, implying the method could lack robustness. Possible over-dispersion in Poisson regression models was not investigated. In addition, multicollinearity among predictors in both general and generalized multiple linear regression models was not assessed. A reason or justification was provided in neither a thesis nor in two dissertations why the non-parametric tests were applied for statistical inferences.

In the survey of statistical methods applied in 1,237 articles published in Acta Scientiarum (Agronomy) from 1998 to 2016 [3], Junior et al. found that approximately 88.2% of articles did not evaluate at least one assumption of the ANOVA, which is consistent with our results. The lack of verification of assumptions in 55.4% of the theses and dissertations in the current study may raise doubts about the reliability of the reported results. The reasons that graduate students fail to test the basic assumptions of ANOVA, regression analysis, and *t*-test may be that they do not know about the tests, their importance, and do not know what constitutes the assumptions. The following inappropriateness of statistical methods are exemplified in some of the theses and dissertations:

- Various types of limitations were observed in 18 theses and eight dissertations related to sampling techniques applied to select samples, such as population units are heterogeneous, but simple random sampling or non-probability sampling techniques were used; sampling units were homogeneous concerning the variable of interest, however, purposive sampling was applied rather than a probability sampling, i.e., simple random sampling; purposive sampling was treated as a probability sampling, and although the layout of study designs suggested multistage sampling, cluster sampling was applied to select samples. Therefore, the methods used could be ineﬃcient.
  Convenience sampling has generated some skepticism among researchers, particularly when it was thought to be a misused sampling technique by some scholars [26] because researchers chose to select data that was conveniently located rather than follow the scientific sampling system [27]. In the current study, we have also noted that theses and dissertations in specific disciplines heavily relied on non-probability sampling techniques.
- R-squared was used as a correlation measure in some of the theses and dissertations, this approach was wrong because squaring a number between -1 and 1 may underestimate the strength of the correlation and ignore the direction of the correlation between variables.
  Although Pearson and Spearman correlation analysis does not show a cause-and-effect relationship, in a few of the theses and dissertations in the current study, the authors applied the method to assess relationships between characteristics. In some cases, authors used to explain phenomena that are uncorrelated or in which one trait does not biologically influence another but has high correlation values. For a cause-and-effect relationship between two characteristics or to assess the biological influence of a character on another character, more accurate statistical techniques like partial correlations and path analysis are appropriate [3], hence the correlation coeﬃcient applied in these theses and dissertations could be irrelevant.
- In some of the theses and dissertations, although the normality assumption was violated, remedial action was not taken; log transformation was done before assessing the normality of a variable, and parallel line assumption for ordinal logistic regression was missing.
  Similar results were found in [2,3], where the authors applied data transformation without checking whether it was necessary. This could be because postgraduate students relied on literature where authors applied transformations to similar data.
- In 17 (12.2%) of the theses/dissertations, the data had repeated measurements or longitudinal data or had nested factors, or had factors, such as season or day. Observations in such data might be correlated, however, these data are modeled incorrectly using either general or generalized linear models including ANOVA ignoring the possible correlation. This leads to invalid confidence intervals for parameters from underestimated standard errors, i.e. incorrect inferences [28].
- In six of the theses/dissertations, multiple responses were assessed whether they were affected by the same factor or treatment separately using one-way ANOVA. However, these response variables might be correlated, and results from one-way ANOVA could be ineﬃcient.
- In some of the theses/dissertations, statistical methods not previously stated in the methodology chapter were reported in the results chapter, and in some cases, results were presented without methods discussed in the methodology chapter or there was a mismatch between methods discussed and results presented.
- Applying multivariate techniques like confirmatory factor analysis or structural equation modeling could have enhanced the statistical analyses’ eﬃciency and allowed for the simultaneous testing of research hypotheses in a few consumer sciences theses and dissertations.

In addition, various theses/dissertations had a number of specific issues, such as:

- there were theses/dissertations with more than one factor with levels that were tested for their effects on response variables, but factors combination with possible interaction effects was not employed.
- Only a second-order interaction in a three-factor factorial experimental design was taken into account; no discussion of a first-order interaction was provided, particularly in cases where the second-order interaction was not statistically significant.
- Multiple comparisons were not carried out in certain instances in one-way or two-way ANOVA when the effects of the treatment, factors, or interaction effects were statistically significant.
- Except for two cases where multiple comparisons were done using the Duncan-multiple range test, in most cases, the least significance difference (LSD) was applied for multiple comparisons.
  In most theses and dissertations in which the LSD test was used, LSD was administered indiscriminately to detect random differences. However, this test should only be used to compare adjacent means in an array where the means are arranged from highest to lowest value, and comparisons between means should be meaningful and planned in advance. Furthermore, with an LSD method, the probability of committing a Type I error increases as the number of pairwise comparisons increases. As a result, there is a greater chance of falsely reporting a significant treatment effect at some point throughout the experiment [29].
- Student’s *t*-test was used for multiple comparisons of the results of the Kruskal–Wallis test and one-way ANOVA.
- Count responses were converted to binary responses and percentages to fit logistic and general binomial regression models, respectively. In some cases, count and ordinal response variables were treated as continuous variables.
- In two theses, quadratic regression models were fitted to the data without any justification or exploratory analysis.

## Conclusion

This study found that over 40% theses/dissertations from those that were assessed had at least one clear methodological error either in the data collection procedure or the data analysis. Almost 95%, that applied a *t*-test did not assume the response variable to be approximately normally distributed. Although the normality assumption was violated in some of the theses and dissertations, remedial action or alternative approach, i.e., a nonparametric approach, was not considered, and correlated data were also incorrectly modelled using generalized linear models, and these could lead to the wrong inferences, such errors in scientific research are very concerning. A lack of knowledge could be the cause of these errors and the use of ineﬃcient statistical methods for data analyses.

Therefore, since postgraduate programs in the university where the current study was conducted involve only research, departments or the college should regularly offer workshops, including statistical and research methods training to improve students’ methodological skills, starting with writing research proposals [4]. In addition, the college should provide university-supported statistical consulting and funding for research work, including statistical analysis. Encourage students to evaluate published articles as part of the workshops and note the advantages and disadvantages of the methodologies used in the articles. This will help them put the skills they have learned in the workshops and training into practice. The authors believe the above recommendation applies to other universities with postgraduate programs conducted only by research and without any course work, as well as those with course work but no statistics and research methods courses in their curriculum. Furthermore, the results of this study imply that MSc/PhD supervisors in the College of Agriculture and Environmental Sciences may need workshops on the latest developed statistical methods and existing advanced methods to assist their students in selecting statistical methodologies for their research. Although the focus of this paper has been on the evaluation of the application of statistical methods in the theses and dissertations submitted to various disciplines in the Agricultural and Environmental Sciences, the type of errors observed here may not be exclusive to these disciplines. Currently, similar studies are going on in other disciplines, and results will be reported elsewhere.
